# Cannabis for pediatric epilepsy: protocol for a living systematic review

**DOI:** 10.1186/s13643-018-0761-2

**Published:** 2018-07-18

**Authors:** Jesse Elliott, Deirdre DeJean, Tammy Clifford, Doug Coyle, Beth Potter, Becky Skidmore, Christine Alexander, Alexander E. Repetski, Bláthnaid McCoy, George A. Wells

**Affiliations:** 1 School of Epidemiology and Public Health, University of Ottawa, 600 Peter Morand Crescent, Ottawa, K1G 5Z3 Canada; 2 Centre for Health Law, Policy and Ethics, University of Ottawa, 57 Louis-Pasteur Private, Ottawa, K1N 6N5 Canada; 3 CADTH, 865 Carling Avenue, Ottawa, QEQ 5Q5 Canada; 4 Ottawa, Canada; 5 Toronto, Canada; 6 Department of Paediatrics, University of Toronto, Toronto, Canada; 7 Division of Neurology, The Hospital for Sick Children Toronto, Toronto, ON Canada; 8 Cardiovascular Research Methods Centre, University of Ottawa Heart Institute, 40 Ruskin Street, Ottawa, K1Y4W7 Canada

**Keywords:** Pediatric epilepsy, Cannabis, Cannabinoids, Cannabidiol, Seizure, Efficacy, Safety, Living systematic review, Meta-analysis

## Abstract

**Background:**

Pediatric epilepsy, including treatment-resistant forms, has a major effect on the quality of life, morbidity, and mortality of affected children. Interest has been growing in the use of medical cannabis as a treatment for pediatric epilepsy, yet there has been no comprehensive review of the benefits and harms of cannabis use in this population. In this systematic review, we will search for, synthesize, and assess the published and gray literature in order to provide usable and relevant information to parents, clinicians, and policy makers.

**Methods:**

We will perform a living systematic review of studies involving the use of cannabis to treat pediatric epilepsy. We will search the published and gray literature for studies involving children with any type of epilepsy taking any form of cannabis. Studies will be selected for inclusion by two independent reviewers. The primary outcome is seizure freedom. Secondary outcomes are seizure frequency, quality of life (child, caregiver), quality and quantity of sleep, status epilepticus, tonic-clonic seizures, death (all-cause, sudden unexpected death in epilepsy), gastrointestinal adverse events (diarrhea, vomiting), and visits to the emergency room. The quality of each included study will be assessed. If data are sufficient in quantity and sufficiently similar, we will conduct pairwise random-effects meta-analysis. We will repeat the literature search every 6 months to identify studies published after the previous search date. Sequential meta-analysis will be performed as necessary to update the review findings.

**Discussion:**

Our review aims to provide a comprehensive and up-to-date summary of the available evidence to inform decisions about the use of cannabis in children with treatment-resistant epilepsy. The results of this review will be of use to parents, clinicians, and policy makers as they navigate this rapidly evolving area.

**Systematic review registration:**

PROSPERO CRD42018084755

**Electronic supplementary material:**

The online version of this article (10.1186/s13643-018-0761-2) contains supplementary material, which is available to authorized users.

## Background

Interest in the use of cannabis for pediatric epilepsy has grown over the last decade, driven in part by media reports of children whose treatment-resistant epilepsy has responded to cannabis [1]. The well-publicized case of Charlotte Figi, whose parents started her on medical cannabis at age 5, is not unique [2], and many parents of children with treatment-resistant epilepsy have reported turning to alternative treatments [3], both with and without the aid of medical professionals. In particular, parents of children with treatment-resistant epilepsy (an inadequate response to two or more adequate trials of antiepileptic drugs [4]), which affects between 28 and 37% of people with epilepsy [5], have expressed great interest in the use of cannabis for the treatment of their children’s epilepsy [3]. Indeed, pediatric treatment-resistant epilepsy has potentially catastrophic consequences, including cognitive delay, behavioral problems, autism, poor quality of life, and early death [6–9].

Despite the recorded medicinal use of cannabis dating back to the second century BCE [10], there has been relatively little research into its effectiveness or safety, likely owing to its illegal status in many jurisdictions. In adults, cannabis has been reported to be effective in the treatment of nausea and vomiting due to chemotherapy, chronic pain, spasticity associated with multiple sclerosis, sleep disorders, and Tourette syndrome, with inconclusive evidence for its use in appetite stimulation in patients with HIV/AIDS, anxiety disorders, and glaucoma [11]. There is relatively little clinical evidence to support the use of cannabis in the treatment of pediatric epilepsy, and there are large discrepancies between the beliefs of health care professionals and the general public with respect to its effectiveness and safety [12]. A 2014 Cochrane review of the use of cannabinoids for the treatment of epilepsy included four small low-quality randomized controlled trials (RCTs), none of which involved children [13]. At the time of this review, the authors concluded that there was insufficient evidence to make reliable conclusions about the efficacy or long-term safety of cannabinoids for the treatment of epilepsy [13]. However, the evidence base in this area is changing rapidly, and recently published studies have suggested a beneficial effect of cannabis in this population [14, 15], although the mechanisms underlying this response are not clear [16].

The use of cannabis in the treatment of pediatric epilepsy is an ideal topic for a living systematic review because it is a priority for decision-making, there is little certainty in the existing evidence base, and there is rapidly accumulating evidence [17]. Living systematic reviews are a relatively new approach to continually updating systematic reviews, with new evidence being incorporated as it becomes available [17]. Unlike traditional systematic review updates, which may be undertaken infrequently, living systematic reviewers search for new studies at a priori-defined intervals and follow a set protocol for determining whether updated analysis and publication are warranted. At present, 37 studies involving the use of various forms of cannabis for the treatment of pediatric epilepsy are registered in ClinicalTrials.gov, suggesting that there will be an abundance of new data in the coming years.

In this living systematic review, we will comprehensively search the published and gray literature for studies that have evaluated the benefits and harms of cannabis for pediatric epilepsy. The results of our review will be of use to parents, clinicians, and policy makers in making treatment decisions for children with epilepsy.

## Methods/design

This systematic review protocol has been submitted to PROSPERO (CRD42018084755) and follows the Preferred Reporting Items for Systematic Reviews and Meta-Analyses Protocols (PRISMA-P) statement (Additional file 1) [18].

### Patient involvement

Two family members of children with epilepsy were involved in selecting the outcome measures for inclusion in this systematic review and are co-authors of this protocol (CA, AR).

### Search strategy

A search of the published and gray literature will be performed by an experienced medical information specialist to identify studies involving the use of cannabis for the treatment of pediatric epilepsy. The search strategy was developed in consultation with an experienced medical information specialist and the research team (Additional file 2). The search will be peer-reviewed by another librarian by use of the Peer Review of Electronic Search Strategies (PRESS) checklist [19]. Using the OVID platform, we will search Ovid MEDLINE®, including Epub Ahead of Print and In-Process & Other Non-Indexed Citations, Embase Classic + Embase, and PsycINFO. We will also search the Cochrane Library on Wiley. The search strategies will use a combination of controlled vocabulary (e.g., “Epilepsy”, “Cannabinoids”, “Medical Marijuana”) and keywords (e.g., “seizure”, “cannabis”, “THC”). Vocabulary and syntax will be adjusted across databases. No date or language restrictions will be imposed. Gray literature will be searched by use of CADTH’s Gray Matters Light [20], Google Scholar, the clinical trials registries ClinicalTrials.gov, and the ICTRP Search Portal of the World Health Organization.

### Eligibility criteria

Studies will be eligible for inclusion if they meet the population, intervention, comparator, and study design criteria described below. Studies will not be selected based on reported outcomes.

#### Population

The population includes children (aged 18 years or younger) with any form of epilepsy. Studies that report mixed populations of children and adults will be included if they report data separately for participants aged less than 19 years or if, based on descriptive statistics, we can determine that at least 60% of patients are aged less than 19 years.

#### Intervention

The intervention is any type of cannabis (synthetic or natural), cannabinol, cannabidiol [CBD], tetrahydrocannabinol [THC], or combinations of these agents. The intervention may be administered by any route (e.g., oral, inhalation), include any strain of cannabis, and any ratio of THC to CBD.

#### Comparators

Eligible comparators include pharmacologic and non-pharmacologic treatments for epilepsy, including antiepileptic drugs, diet therapy, vagus nerve stimulation, and resective brain surgery. Comparators may also include placebo, usual care, or no treatment.

#### Outcomes

The primary outcome is seizure freedom. Secondary outcomes are seizure frequency, quality of life (child, caregiver), improvement or worsening of sleep, status epilepticus, tonic-clonic seizures, death (all-cause, sudden unexpected death in epilepsy), gastrointestinal adverse events (diarrhea, vomiting), and visits to the emergency room. Study eligibility will not be evaluated based on reported outcomes.

#### Study designs

Eligible study designs are RCTs (including cross-over and N-of-1 trials), quasi-randomized controlled trials, controlled before-after, non-randomized controlled trials, historically or concurrently controlled cohort series [prospective or retrospective], case-control studies, uncontrolled cohort studies, case series involving at least five patients, and cross-sectional studies. Conference abstracts and clinical trial registrations will be eligible for inclusion.

### Screening and selection procedure

Two independent reviewers will screen the title and abstract of each identified record and assess the eligibility against the above criteria. The full text of any record deemed potentially relevant by at least one reviewer will be examined in detail and evaluated for eligibility by two independent reviewers. Disagreements on eligibility will be resolved by discussion. Studies will not be excluded based on treatment duration, and no language exclusions will be applied. All screening and data extraction will be performed using standardized and piloted forms in Distiller SR (Evidence Partners).

### Data extraction

Data will be extracted by one reviewer and checked for accuracy by a second reviewer. Data to be extracted includes study characteristics (e.g., first author, year of publication, study design, country of study), participant characteristics (e.g., age, sex, epilepsy syndrome, ethnicity, comedications, comorbidities), intervention and comparator details (e.g., type of treatment, dose, route of administration, duration), and outcome data at the longest duration of follow-up. Event counts will be extracted for dichotomous outcomes (seizure freedom, improvement or worsening of sleep, status epilepticus, deaths, diarrhea, vomiting, visits to the emergency room). Data for continuous outcomes (seizure frequency, quality of life, quality and/or quantity of sleep) will be extracted as mean or median change from baseline or difference between groups after treatment. If available, the number of participants who achieve at least a 50% reduction in seizures from baseline will be extracted. Estimates of variability (e.g., standard deviation, standard error, 95% confidence intervals) will be extracted. First-period data as well as end-of-study effects will be extracted from cross-over studies. Additional information will be extracted, including the number of cases and controls (case-control studies), adjusted odds ratios, relative risk or hazard ratios, and 95% confidence intervals, as reported by the study authors. No data will be extracted from studies that do not present study-specific data (e.g., odds ratios, 95% confidence intervals) or provide sufficient information for calculation of an outcome measure (event counts and denominators).

### Quality and risk of bias assessment

The quality of included studies that report at least one outcome of interest will be assessed by use of the Cochrane Collaboration’s Risk of Bias tool for RCTs [21]. The Institute of Health Economics Quality Appraisal Checklist for Case Series Studies [22], or SIGN50 [23] will be used for other study designs as appropriate. Other quality assessment tools will be identified as needed depending on the designs of the included studies. Quality will be assessed by two independent reviewers, and disagreements will be resolved by discussion.

### Data synthesis

First, we will provide a descriptive summary of study selection, study and patient characteristics, and the results of the quality assessment.

Second, we will assess whether the data are sufficient in quantity and sufficiently homogeneous to be pooled via random-effects meta-analyses (i.e., minimal clinical, methodological, statistical heterogeneity). We will assess clinical heterogeneity by examining the patient characteristics of the included studies and methodological heterogeneity by assessing the study characteristics. Statistical heterogeneity will be assessed by use of the *I*^2^ statistic. If substantial heterogeneity is present (*I*^2^ > 75%), we will explore this by subgroup analyses. At *I*^2^ values above 75%, pooled data will not be reported, and descriptive summaries of the study-level findings will be presented. The primary analysis will involve data from RCTs involving children (at least 80% of participants aged less than 19 years) with any type of epilepsy, and data from other study designs will be evaluated in secondary analyses if deemed appropriate. Other secondary analyses will involve (a) studies including participants with treatment-resistant epilepsy or (b) studies in which at least 60% of the population is aged less than 19 years. If data are sufficient and sufficiently similar, meta-analysis will be performed by use of RevMan (v.5.3; Cochrane Collaboration). If there are insufficient data for meta-analysis, we will report descriptive summaries for each outcome. The findings of conference abstracts will be summarized descriptively.

### Additional analyses

If sufficient data are available, sensitivity analyses will be performed to test the robustness of the estimates. For example, we will look at the impact of excluding studies with high risk of bias or poor methodological quality. We will also assess the impact of comedications; study location; and cannabis strain, dose, level of THC, or ratio of THC to CBD; or route of administration, depending on data availability. Subgroup analyses will investigate the effect of epilepsy syndrome (e.g., Dravet syndrome, Lennox-Gastaut syndrome), age, and sex. Publication bias will be assessed by visual inspection of funnel plots for outcomes that have data from at least ten studies [24].

### Quality of the evidence

The quality of the evidence will be assessed for each outcome by use of the Grades of Recommendations, Assessment, Development and Evaluation (GRADE) Working Group method [25].

### Update plan

In keeping with current recommendations for the conduct of living systematic reviews [17], the literature search will be repeated at regular a priori-defined intervals to identify newly available studies. Published and gray literature searches will be updated every 6 months, and any records identified after the date of the last update will be screened for inclusion as described above. This frequency of updating was chosen to maximize the visibility of new information for decision-makers but to minimize the resources required [17]. There are three potential update scenarios: (1) no new evidence is located, (2) new evidence is located but is not sufficient to trigger updated analysis and publication, or (3) new evidence is located and new analysis and dissemination is required [17]. If new studies that meet the eligibility requirements are located, the reviewer team will decide, in consultation with a clinical expert (BM), whether updated analyses are required, with consideration of balancing the needs of decision-makers with the available resources [17]. If required, meta-analyses will be updated by use of trial sequential analyses (type I error, 0.05; power, 80%; assumed effect size, moderate Cohen’s effect size [0.5 standard deviations]) for living systematic reviews [26].

## Discussion

Pediatric epilepsy has a major impact on the quality of life for patients and their families. Interest in the use of cannabis as a treatment for pediatric epilepsy and, in particular, for treatment-resistant epilepsy has grown over the last decade, driven in part by media reports of children whose treatment-resistant epilepsy has responded to cannabis after the failure of conventional treatments [1]. This living systematic review will provide a comprehensive summary of the current evidence for the use of cannabis for the treatment of pediatric epilepsy and will incorporate new evidence as it becomes available. The results will be of use to decision-makers, including parents of affected children, clinicians, and policy makers.

## Additional files

Additional file 1: PRISMA-P checklist. (DOC 97 kb)

Additional file 2: Search strategy. (DOCX 18 kb)

## Abbreviations

CBD: Cannabidiol
RCT: Randomized controlled trial
THC: Tetrahydrocannabinol
