# Supplementary material for: Cannabis for pediatric epilepsy: protocol for a living systematic review
Source: Syst Rev. 2018 Jul 18;7:95. doi: 10.1186/s13643-018-0761-2 (PMC6052624; doi:10.1186/s13643-018-0761-2)
Supplement: Supplementary file 2 — Search strategy. (DOCX 18 kb) [file 13643_2018_761_MOESM2_ESM.docx]

Ovid Multifile: Database: Embase Classic+Embase <1947 to 2017 December 13>, Ovid MEDLINE(R) ALL <1946 to December 14, 2017>, PsycINFO <1806 to December Week 2 2017>

Search Strategy:

--------------------------------------------------------------------------------

1 exp Epilepsy/ (413158)

2 (epileps* or epilept*).tw,kf. (354209)

3 seizure*.tw,kf. (308442)

4 convulsi*.tw,kf. (73122)

5 falling sickness*.tw,kf. (61)

6 comitial disease*.tw,kf. (3)

7 (petit mal or grand mal or absence status).tw,kf. (8405)

8 Landau-Kleffner Syndrome*.tw,kf. (1213)

9 Lennox Gastaut Syndrome*.tw,kf. (3397)

10 Dravet Syndrome*.tw,kf. (2044)

11 West syndrome*.tw,kf. (2852)

12 Doose syndrome*.tw,kf. (167)

13 Ohtahara syndrome*.tw,kf. (499)

14 Sturge-Weber Syndrome/ (3596)

15 ((sturge* or weber) adj2 (disease* or syndrome*)).tw,kf. (8090)

16 (myoclonic encephalopath* or action myoclonus-renal failure syndrome* or atypical inclusion-body disease* or biotin-responsive encephalopath* or haw river syndrome* or may white syndrome* or myoclonus-nephropathy syndrome* or naito oyanagi disease*).tw,kf. (776)

17 SMEI.tw,kf. (461)

18 (MERRF or fukuhara disease* or fukuhara disorder* or myoencephalopathy ragged-red fiber disease* or myoencephalopathy ragged-red fibre disease*).tw,kf. (1137)

19 Lafora.tw,kf. (1430)

20 ((Unverricht adj1 Lundborg) or Baltic Myoclonus or Unverricht disease* or Unverricht syndrome*).tw,kf. (735)

21 ((infantile or nodding) adj2 spasm?).tw,kf. (6276)

22 ((flexor or "in flexion") adj2 spasm?).tw,kf. (332)

23 ((lightning or salaam) adj2 attack?).tw,kf. (16)

24 hypsarrhythmi*.tw,kf. (2063)

25 or/1-24 [EPILEPSY] (627889)

26 Adolescent/ (3482184)

27 exp Child/ (4584752)

28 exp Infant/ (2191998)

29 (boy or boys or girl or girls or infant* or infanc* or baby or babies or child* or toddler* or preschool* or pre-school* or school-age* or adolescen* or teen or teens or teenager* or youth or youths or highschool* or high-school*).tw,kf. (5312514)

30 (newborn* or neonat*).tw,kf. (874968)

31 (pediatric* or paediatric*).tw,kf. (811013)

32 or/26-31 (9306058)

33 25 and 32 [PEDIATRIC EPILEPSY] (223050)

34 Cannabis/ (43738)

35 exp Cannabinoids/ (76752)

36 Medical Marijuana/ (1233)

37 Marijuana Smoking/ (6987)

38 ("c.indica" or cannabi* or bhang or cannador or charas or eucannabinolide* or ganja or ganjah or hash or hashish or hemp or marihuana* or marijuana*).tw,kf. (112741)

39 (epidiolex or gwp 42003p or gwp42003p or nabidiolex).tw,kf. (79)

40 (dronabinol or thc or tetrahydrocannabinol* or ea 1477 or ea1477 or marinol or qcd 84924 or syndros or tetranabinex).tw,kf. (23589)

41 (deltanyne or "abbott 40566" or namisol or dronabinolum or "QCD 84924" or "CCRIS 4726").tw,kf. (22)

42 or/34-41 (144274)

43 33 and 42 [CANNABIS - PEDIATRIC EPILEPSY] (670)

44 exp Animals/ not (exp Animals/ and Humans/) (16160986)

45 43 not 44 [ANIMAL-ONLY REMOVED] (453)

46 45 use medall (185)

47 exp epilepsy/ (413158)

48 (epileps* or epilept*).tw,kw. (359359)

49 seizure*.tw,kw. (310739)

50 convulsi*.tw,kw. (74278)

51 falling sickness*.tw,kw. (64)

52 comitial disease*.tw,kw. (3)

53 (petit mal or grand mal or absence status).tw,kw. (8283)

54 Landau-Kleffner Syndrome*.tw,kw. (1248)

55 Lennox Gastaut Syndrome*.tw,kw. (3450)

56 Dravet Syndrome*.tw,kw. (2094)

57 West syndrome*.tw,kw. (2980)

58 Doose syndrome*.tw,kw. (181)

59 Ohtahara syndrome*.tw,kw. (513)

60 sturge-weber syndrome/ (3596)

61 ((sturge* or weber) adj2 (disease* or syndrome*)).tw,kw. (8213)

62 (myoclonic encephalopath* or action myoclonus-renal failure syndrome* or atypical inclusion-body disease* or biotin-responsive encephalopath* or haw river syndrome* or may white syndrome* or myoclonus-nephropathy syndrome* or naito oyanagi disease*).tw,kw. (785)

63 SMEI.tw,kw. (485)

64 (MERRF or fukuhara disease* or fukuhara disorder* or myoencephalopathy ragged-red fiber disease* or myoencephalopathy ragged-red fibre disease*).tw,kw. (1167)

65 Lafora.tw,kw. (1443)

66 ((Unverricht adj1 Lundborg) or Baltic Myoclonus or Unverricht disease* or Unverricht syndrome*).tw,kw. (740)

67 ((infantile or nodding) adj2 spasm?).tw,kw. (6422)

68 ((flexor or "in flexion") adj2 spasm?).tw,kw. (332)

69 ((lightning or salaam) adj2 attack?).tw,kw. (16)

70 hypsarrhythmi*.tw,kw. (2094)

71 or/47-70 [EPILEPSY] (630155)

72 juvenile/ (38711)

73 exp adolescent/ (3482352)

74 exp child/ (4584752)

75 (boy or boys or girl or girls or infant* or infanc* or baby or babies or child* or toddler* or preschool* or pre-school* or school-age* or adolescen* or teen or teens or teenager* or youth or youths or highschool* or high-school*).tw,kw. (5321220)

76 (newborn* or neonat*).tw,kw. (870009)

77 (pediatric* or paediatric*).tw,kw. (831059)

78 or/72-77 (9154566)

79 71 and 78 [PEDIATRIC EPILEPSY] (221973)

80 cannabis/ (43738)

81 exp cannabinoid/ (63688)

82 medical cannabis/ (1625)

83 exp "cannabis use"/ (7317)

84 ("c.indica" or cannabi* or bhang or cannador or charas or eucannabinolide* or ganja or ganjah or hash or hashish or hemp or marihuana* or marijuana*).tw,kw. (113719)

85 (epidiolex or gwp 42003p or gwp42003p or nabidiolex).tw,kw. (79)

86 (dronabinol or thc or tetrahydrocannabinol* or ea 1477 or ea1477 or marinol or qcd 84924 or syndros or tetranabinex).tw,kw. (23862)

87 (deltanyne or "abbott 40566" or namisol or dronabinolum or "QCD 84924" or "CCRIS 4726").tw,kw. (22)

88 or/80-87 (143934)

89 79 and 88 [CANNABIS - PEDIATRIC EPILEPSY] (678)

90 exp animal experimentation/ or exp animal model/ or exp animal experiment/ or nonhuman/ or exp vertebrate/ (48509001)

91 exp human/ or exp human experimentation/ or exp human experiment/ (37777771)

92 90 not 91 (10732950)

93 89 not 92 [ANIMAL-ONLY REMOVED] (631)

94 93 use emczd [EMBASE RECORDS] (399)

95 exp Epilepsy/ (413158)

96 Status Epilepticus/ (17946)

97 (epileps* or epilept*).tw. (350570)

98 seizure*.tw. (307670)

99 convulsi*.tw. (71469)

100 falling sickness*.tw. (59)

101 comitial disease*.tw. (3)

102 Grand Mal Seizures/ (196)

103 Petit Mal Seizures/ (127)

104 (petit mal or grand mal or absence status).tw. (8218)

105 Landau-Kleffner Syndrome*.tw. (1210)

106 Lennox Gastaut Syndrome*.tw. (3379)

107 Dravet Syndrome*.tw. (2019)

108 West syndrome*.tw. (2812)

109 Doose syndrome*.tw. (160)

110 Ohtahara syndrome*.tw. (494)

111 ((sturge* or weber) adj2 (disease* or syndrome*)).tw. (8041)

112 (myoclonic encephalopath* or action myoclonus-renal failure syndrome* or atypical inclusion-body disease* or biotin-responsive encephalopath* or haw river syndrome* or may white syndrome* or myoclonus-nephropathy syndrome* or naito oyanagi disease*).tw. (775)

113 SMEI.tw. (456)

114 (MERRF or fukuhara disease* or fukuhara disorder* or myoencephalopathy ragged-red fiber disease* or myoencephalopathy ragged-red fibre disease*).tw. (1127)

115 Lafora.tw. (1421)

116 ((Unverricht adj1 Lundborg) or Baltic Myoclonus or Unverricht disease* or Unverricht syndrome*).tw. (730)

117 ((infantile or nodding) adj2 spasm?).tw. (6224)

118 ((flexor or "in flexion") adj2 spasm?).tw. (332)

119 ((lightning or salaam) adj2 attack?).tw. (15)

120 hypsarrhythmi*.tw. (2024)

121 or/95-120 [EPILEPSY] (626451)

122 (boy or boys or girl or girls or infant* or infanc* or baby or babies or child* or toddler* or preschool* or pre-school* or school-age* or adolescen* or teen or teens or teenager* or youth or youths or highschool* or high-school*).tw. (5256005)

123 (newborn* or neonat*).tw. (860199)

124 (pediatric* or paediatric*).tw. (800591)

125 or/122-124 (6005033)

126 121 and 125 [PEDIATRIC EPILEPSY] (158962)

127 exp Cannabis/ (46269)

128 exp Cannabinoids/ (76752)

129 Marijuana Usage/ (2514)

130 ("c.indica" or cannabi* or bhang or cannador or charas or eucannabinolide* or ganja or ganjah or hash or hashish or hemp or marihuana* or marijuana*).tw. (112358)

131 (epidiolex or gwp 42003p or gwp42003p or nabidiolex).tw. (79)

132 (dronabinol or thc or tetrahydrocannabinol* or ea 1477 or ea1477 or marinol or qcd 84924 or syndros or tetranabinex).tw. (23463)

133 (deltanyne or "abbott 40566" or namisol or dronabinolum or "QCD 84924" or "CCRIS 4726").tw. (22)

134 or/127-133 (143233)

135 126 and 134 [CANNABIS - PEDIATRIC EPILEPSY] (510)

136 exp Animals/ not (exp Animals/ and Humans/) (16160986)

137 135 not 136 [ANIMAL-ONLY REMOVED] (333)

138 137 use medall,emczd (279)

139 137 not 138 [PSYCINFO RECORDS] (54)

140 46 or 94 or 139 [ALL DATABASES] (638)

141 remove duplicates from 140 (441) [TOTAL UNIQUE RECORDS]

142 141 use medall [MEDLINE UNIQUE RECORDS] (138)

143 141 use emczd [EMBASE UNIQUE RECORDS] (270)

144 141 not (142 or 143) [PSYCINFO UNIQUE RECORDS] (33)
